# *Lactobacillus paracasei* feeding improves immune control of influenza infection in mice

**DOI:** 10.1371/journal.pone.0184976

**Published:** 2017-09-20

**Authors:** Nouria Belkacem, Nicolas Serafini, Richard Wheeler, Muriel Derrien, Lilia Boucinha, Aurélie Couesnon, Nadine Cerf-Bensussan, Ivo Gomperts Boneca, James P. Di Santo, Muhamed-Kkeir Taha, Raphaëlle Bourdet-Sicard

**Affiliations:** 1 Institut Pasteur, Invasive Bacterial Infections Unit, Paris, France; 2 Bioaster, Paris, France; 3 Innate Immunity Unit, Institut Pasteur, Paris, France; 4 Inserm U1223, Paris, France; 5 Institut Pasteur, Unité Biologie et génétique de la paroi bactérienne, Dept. Microbiologie, Paris, France; 6 Institut National de la santé et de la Recherche Médicale (INSERM), Paris, France; 7 DanoneNutricia Research, Palaiseau, France; 8 INSERM, U1163, Laboratory of Intestinal Immunity, Université Paris Descartes-Sorbonne Paris Cité and Institut Imagine, Paris, France; Mayo Clinic Minnesota, UNITED STATES

## Abstract

Respiratory tract infections such as flu cause severe morbidity and mortality and are among the leading causes of death in children and adults worldwide. Commensal microbiota is critical for orchestrating tissue homeostasis and immunity in the intestine. Probiotics represent an interesting source of immune modulators and several clinical studies have addressed the potential beneficial effects of probiotics against respiratory infections. Therefore, we have investigated the mechanisms of protection conferred by *L*. *paracasei* CNCM I-1518 strain in a mouse model of influenza infection. Notably, local myeloid cells accumulation is generated in the lungs after seven days feeding with *L*. *paracasei* prior to viral infection. *L*. *paracasei*-fed mice showed reduced susceptibility to the influenza infection, associated with less accumulation of inflammatory cells in the lungs, faster viral clearance and general health improvement. Interestingly, *Allobaculum* was significantly increased in *L*. *paracasei*-fed mice 7 days after influenza infection, even if the gut microbiota composition was not altered overall. *L*. *paracasei*-purified peptidoglycan partially recapitulated the protective phenotype observed with the entire bacteria. Collectively, our results demonstrate that oral consumption of *L*. *paracasei* CNCM I-1518 modulates lung immunity was associated with an improved control of influenza infection. These results further extend the beneficial role for certain lactobacilli to alleviate the burden of respiratory tract infections.

## Introduction

Influenza virus is a major source of severe viral respiratory infections in adults, causing annual epidemics that result in important morbidity and mortality. Major pandemics throughout the 20^th^ century have killed more than 100 million people [1]. The constant threat of the emergence of a novel influenza subtype represents a global danger and necessitates constant surveillance. Vaccination is the principal measure to control influenza epidemics. Unfortunately, subjects who present a higher risk of severe complications after influenza infection (such as in elderly) are also those who do not respond efficiently to conventional influenza vaccines compared to healthy adults [2, 3].

Interestingly, commensal bacteria have been reported as essential for immune homeostasis and functions. In fact, respiratory tract dendritic cells were not able to migrate to the draining lymph node and prime T-cell responses in antibiotic-treated mice after influenza infection [4]. Indeed, the microbiota provides stimulatory and maturation signals to immune system, leading to protection against potential pathogens and microbiota disorders [5].

Probiotics, specifically lactobacilli and bifidobacteria, have been extensively studied for their protective effects toward respiratory infections in children, young adults and the elderly. Recent meta-analyses and systematic reviews indicated that probiotic consumption could reduce incidence, duration, or severity of respiratory infections [6–8]. Notably, daily consumption of a fermented dairy product containing *L*. *paracasei* CNCM I-1518 was reported to reduce the incidence of common gastrointestinal and respiratory infections in children [9] and to lower the risk of common infections in stressed adults, associated with an increase of NK cells during infection [10]. The consumption of the same fermented milk was also associated with a decreased duration of upper respiratory tract infections in elderly subjects [11]. The mechanisms behind such health improvement of respiratory infections induced by oral consumption of lactobacilli are not clearly understood.

Because the underlying mechanism cannot be addressed in human trials, the development of a murine experimental flu infection model using an influenza A strain offers a well-defined system to investigate the impact of lactobacilli and their bacterial components on the control of viral infection as well as interactions with microbiota [12].

Interestingly, the anti-inflammatory capacity of cell-wall components from selected lactobacilli was shown to be crucial for the regulation of intestinal inflammation, occurring through host recognition of e bacterial peptidoglycan and NOD2 signalling [13].

In this report, we present evidences that the *L*. *paracasei* CNCM I-1518 strain provides a critical benefit in regulating airway tissue integrity and in orchestrating the pulmonary immune defense after experimental infection with influenza virus.

## Results

### Effects of *L*. *paracasei* on influenza-infected mice health status

**Fig 1:**
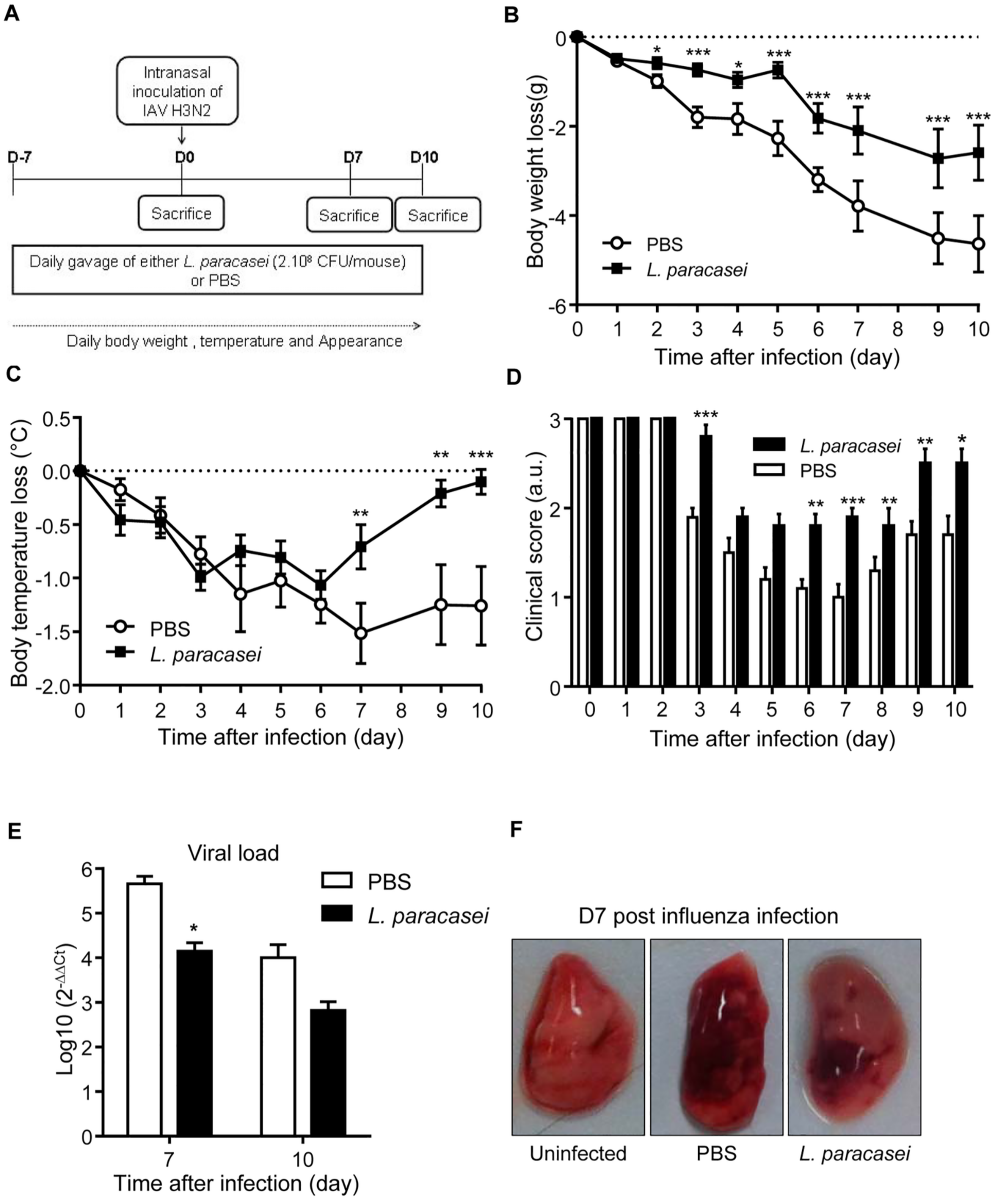
Effects of *L*. *paracasei* consumption on health status of influenza-infected mice. (A) An overview of the experimental scheme; (B) Body weight loss that was expressed in comparison to day 0 of viral infection for each mouse. We scored each individual mouse in comparison to its weight at day 0 as no significant differences were observed prior to viral infection. (C) Temperature loss expressed as the difference between values at D0 in mice after influenza infection with 260 pfu; (D) Score appearance of mice after influenza infection. Results are expressed as mean ± SEM for each group (n = 31). (*p<0.05, **P<0.01, ***P<0.001, **** P<0.0001). (E) Viral load measured with RTq-PCR at D7 or D10 post influenza infection in the lungs of mice fed with either *L*. *paracasei* (N = 15) or with PBS (N = 16). (F). Hemorrhagic lesion in the lungs of *L*. *paracasei*- or PBS fed mice at day 7 post influenza infection compared with non-infected mice. Data are means ± SEM of each group of mice. (*p<0.05).

To assess the impact of *L*. *paracasei* CNCM I-1518 oral administration on resistance toward influenza infection, we first focused on the health status evolution of mice receiving either *L*. *paracasei* or control (Phosphate Saline Buffer: PBS) 7 days prior to viral infection. Mice were infected intranasally with influenza A H3N2 variant virus at day 0 and followed up to 10 days after viral infection (Fig 1A).

As expected, prior to influenza intranasal infection, the consumption of *L*. *paracasei* did not induce any significant changes on weight, temperature and appearance of mice (S1 Fig). Moreover, no histological differences were evidenced in the lungs prior to the influenza infection (S2A Fig). After infection with influenza virus, infected control mice rapidly lost body weight (Fig 1B) and body temperature (Fig 1C) and developed pathological appearance (Fig 1D). The analysis of viral load by RT-PCR revealed that *L*. *paracasei*-fed mice had lower influenza loads after 3, 7 and 10 days of viral infection (Fig 1E and S2B Fig). Moreover, fewer hemorrhagic lesions were observed in the lungs of *L*. *paracasei*-fed mice compared with the control group, 7 days after influenza infection (Fig 1F and S2C Fig). Collectively, these results suggest that consumption of *L*. *paracasei*, initiated before influenza infection was associated with better health status in mice.

In order to better characterize whether the observed *L*. *paracasei*-induced health improvement could be due to a local protective effect against influenza-induced gut mucosa damage, histological analyses were conducted on gut tissues before and after influenza infection. Before infection, no histological differences were evidenced in the small intestine of mice fed either with PBS or *L*. *paracasei* (S3a and S3b Fig respectively). Seven days after intranasal H3N2 viral infection, no alteration of intestinal tissue, such as atrophy of villi or lymphocytic infiltration could be detected in either PBS or *L*. *paracasei*-treated mice (S2c and S3d Figs respectively). The selected images were taken from the jejunum region, but similar results were obtained both in the duodenum and ileum. In addition, no diarrhea was observed upon infection in both gavage conditions. Altogether, these results demonstrate that the mouse-adapted influenza cannot affect the intestinal barrier and that the *L*. *paracasei* induced health status improvement was not linked to protection against intestinal damage. Moreover, we tested the impact of two other *Lactobacillus* strains in the in the influenza H3N2 infection model. In these conditions, *L*. *paracasei* CNCM I-3689 and *L*. *rhamnosus* CNCM I-3690 did not promote improvement in body weight loss (S4a Fig), nor a reduction on the viral load in the lungs (S4b Fig). Altogether, the protective effects induced by *L*. *paracasei* CNCM I-1518 in the influenza H3N2 experimental model appeared to be strain specific.

### Effects of consumption of *L*. *paracasei* on cytokine profiles in the lungs

**Fig 2:**
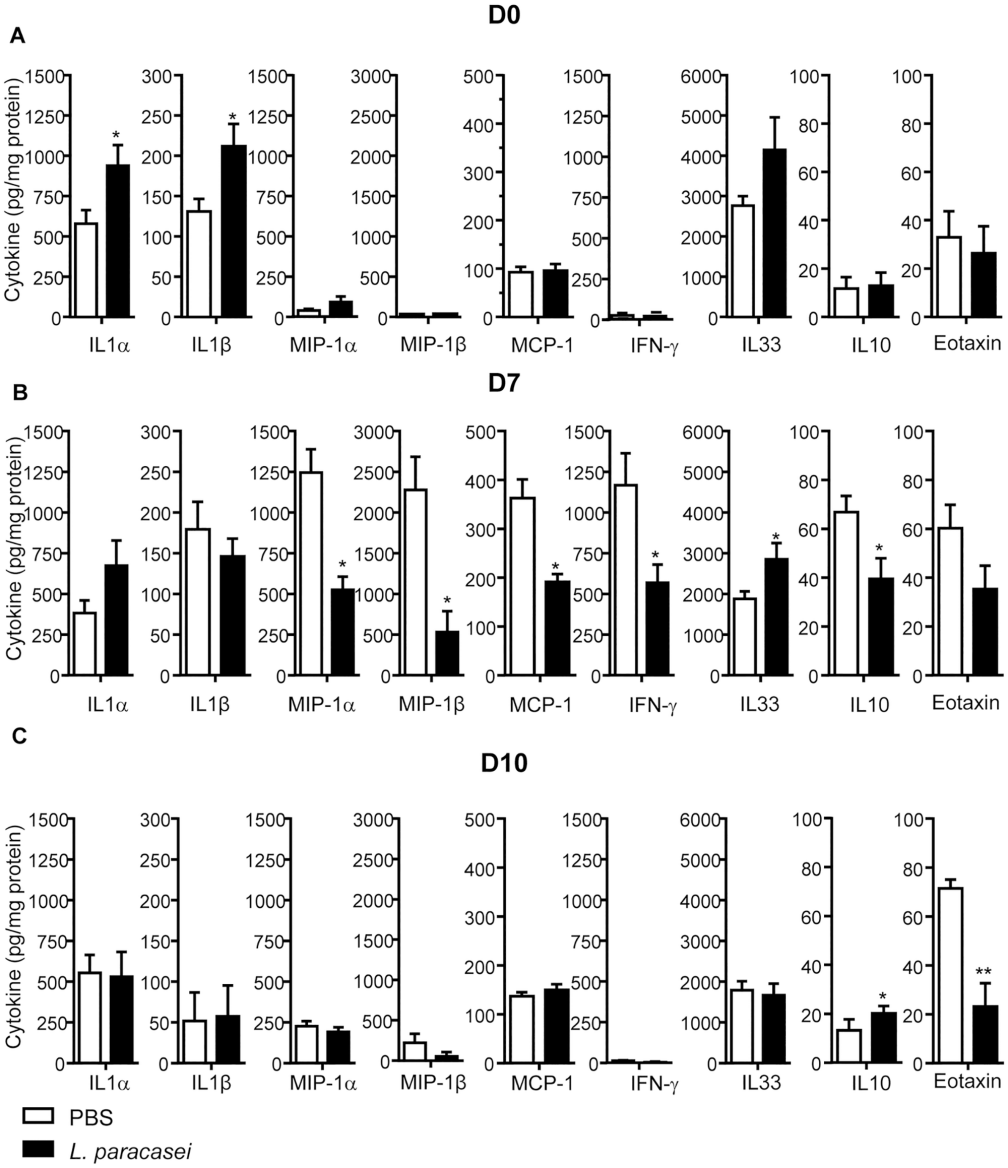
Effects of consumption of *L*. *paracasei* on cytokine profiles, compared with the control group. A: day 0, B: day 7, C: day 10 after influenza infection. Results are expressed as mean ± SEM for each group (n = 8).

To understand the beneficial effect of *L*. *paracasei*, we next investigated the impact of the lactobacilli on the inflammatory status. In fact, the balance of pro and anti-inflammatory cytokines is essential to promote balanced immune responses during influenza infection [14]. Before influenza infection (day 0, D0), we observed significantly higher levels of pro-inflammatory cytokines IL-1α and IL-1β in *L*. *paracasei*-fed mice compared to PBS-fed mice (Fig 2A). We did not observe any significant difference in the others cytokines tested between the two groups. Interestingly, this trend was reversed at D7 after influenza infection with less inflammatory cytokines such as Mip-1α, Mip-1β, IFN-γ and MCP-1 in *L*. *paracasei*–fed mice compared to control mice. However, at D7, IL-33 showed significantly higher levels in the lungs of *L*. *paracasei*-fed mice compared to the control group (Fig 2B). Ten days after influenza infection, we observed more IL-10 and less Eotaxin in mice that consumed *L*. *paracasei* compared with control group (Fig 2C). We did not observe significant differences in the others cytokines tested between the two groups.

These results collectively indicate that oral administration of *L*. *paracasei* modified the pro- and anti-inflammatory cytokine releases in the lungs before and after influenza infection.

### Effect of consumption of *L*. *paracasei* on the recruitment and activation of immune cells in the lungs

The innate and adaptive immune compartments play a key role in both the initiation and the development of influenza virus-mediated inflammation [15]. To further assess the effect of *L*. *paracasei* on immune response, total cell counts in the lungs were quantified before (D0) and after influenza infection (D7 and D10). This quantification showed 2.5-fold higher counts of total cells in the lungs at D0 in *L*. *paracasei*–fed mice than in the control group (S5a Fig). Total cell counts in the lungs were largely increased in both groups after viral infection, and no longer differed between groups at D7 and D10 (S5a Fig). The resident immune cells profile was similar at D0 both in the lungs from *L*. *paracasei*–fed mice and from control mice (S5b Fig). The substantial infiltration of cells in the lungs of *L*. *paracasei*-treated mice was not due to an influx of *L*. *paracasei* in the lungs, as confirmed by PCR targeting specific *L*. *paracasei* CNCM I-1518 CRISPR (clustered regularly interspaced short palindromic repeats) (S5c Fig).

The increase of total cells in the lung at D0 was strain specific, as the two others *lactobacillus* strains tested in the same conditions (*L*. *paracasei* CNCM I-3689 and *L*. *rhamnosus* CNCM I-3690) did not induced cells recruitment in lungs prior to influenza infection (S4c Fig).

**Fig 3:**
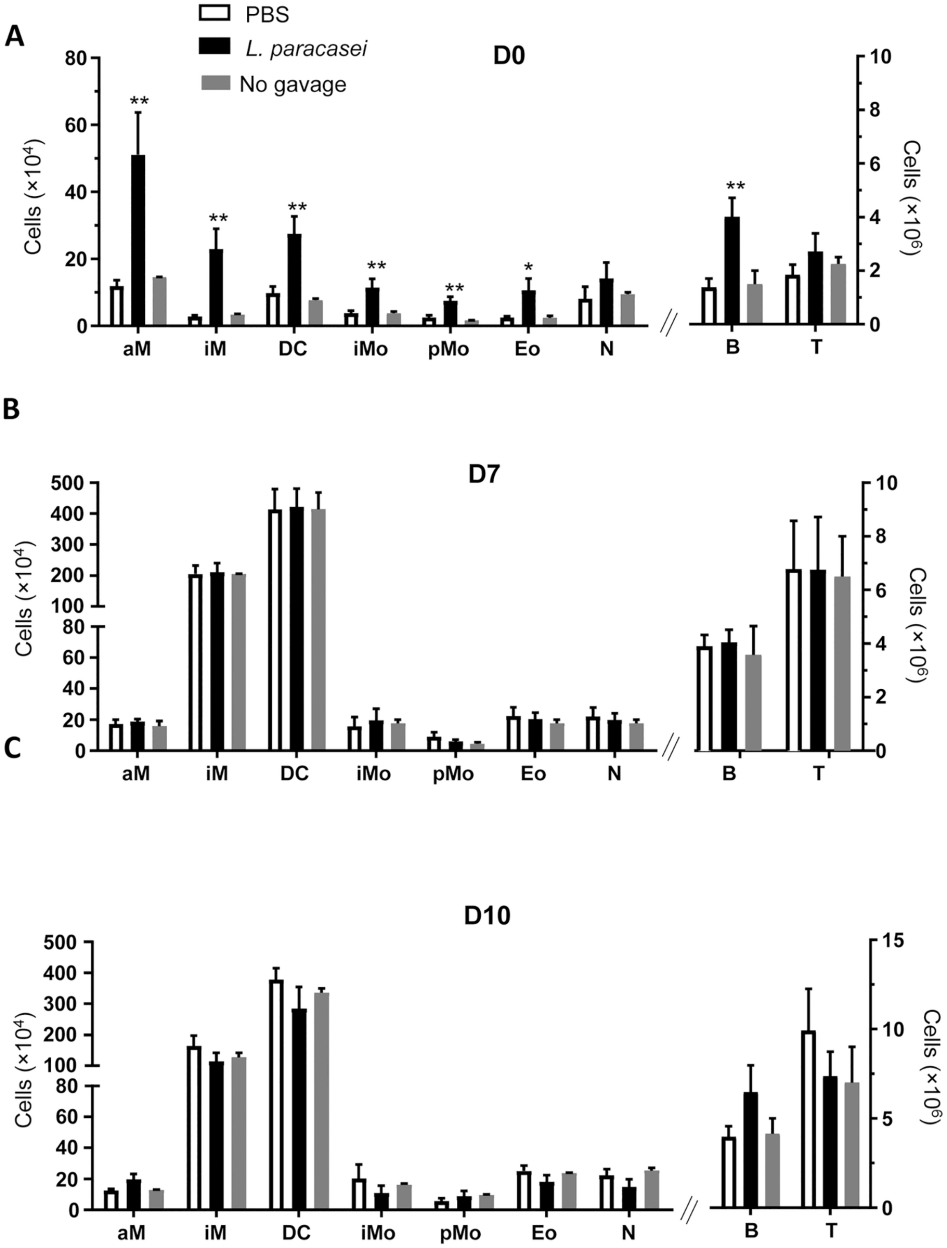
Effect of consumption of *L*. *paracasei* (N = 30) on the recruitment of immune cells in the lungs at different days. A: day 0, B: day 7, C: day 10 after influenza infection, compared with the control group (Mice gavaged with PBS and infected by influenzae virus) (N = 30). Or to mice that were only infected with influenzae virus but with no gavage (N = 5) aM: Alveolar Macrophage, iM: Interstitial Macrophage, DC: Dendritic cells, iM: Inflammatory Monocyte, pM: Patrolling monocytes, Eo: Eosinophils, N: Neutrophils, B: B cells, T: T cells. Results are expressed as mean ± SEM for each group. (*p<0.05, **P<0.01).

Flow cytometry analysis revealed a major increase in the number of all tissue resident or circulatory myeloid cells (alveolar macrophages, interstitial macrophages, dendritic cells, eosinophils, inflammatory monocytes, patrolling monocytes) and B cells after *L*. *paracasei* gavage and before the viral infection, when compared to the control group (Fig 3A). No difference was observed after infection (D7 or D10; Fig 3B and 3C). The effects observed after 7 days of gavage was not due to the “physical action of gavage”. Mice that were only infected by influenzae virus but with no gavage showed no significant differences in immune cells when compared to those that were gavaged by PBS (Fig 3). Moreover, these two groups of mice (not gavaged and PBS-gavaged) did not show any difference in viral load at days 7 after flu infection (S6 Fig).

**Fig 4:**
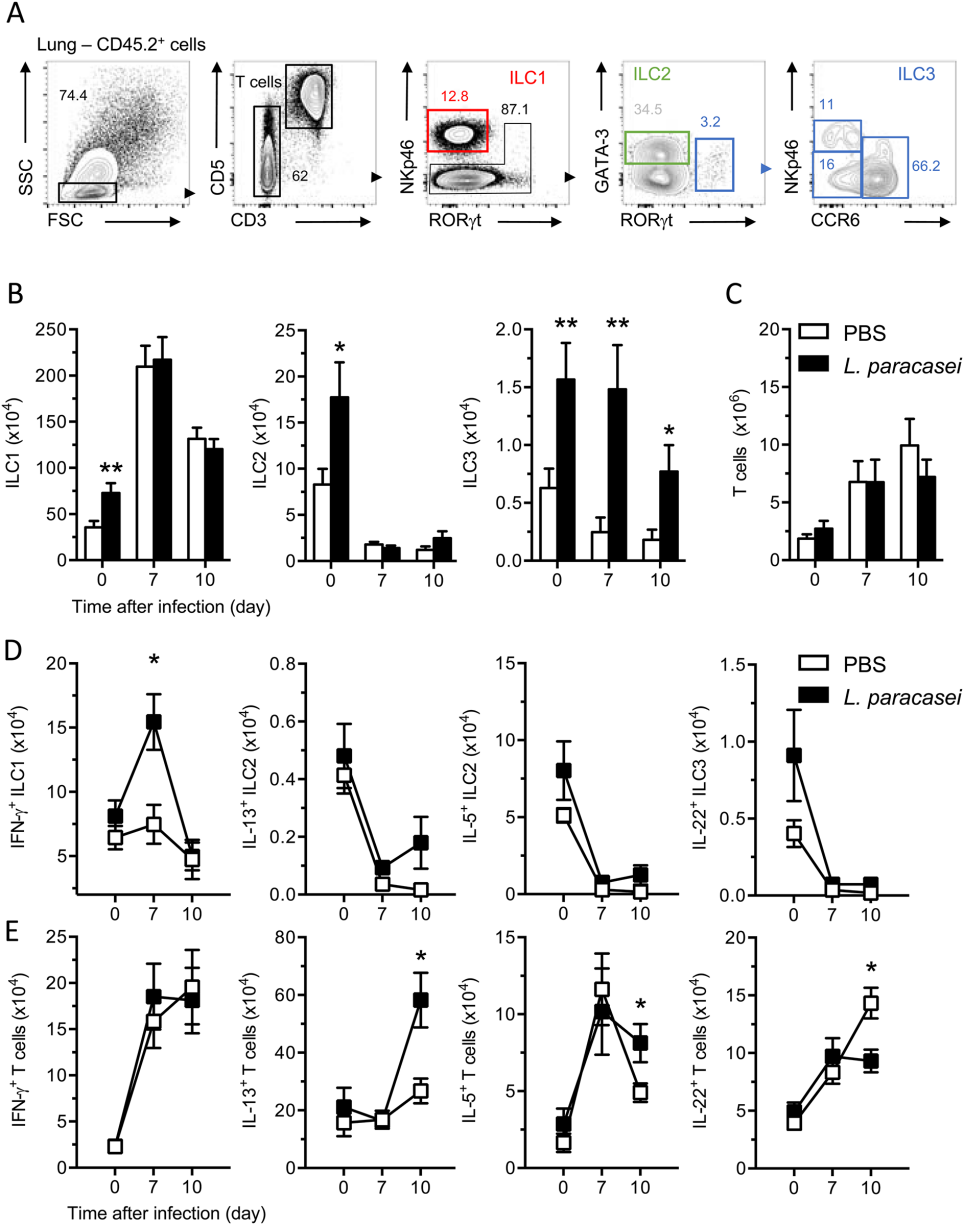
Effects of consumption of *L*. *paracasei* on ILCs and T effector cells. (A). gating strategy for FACS analysis of mouse lung. ILC1 were gated on CD45^+^CD3^-^CD5^-^RORγt^-^NKp46^+^, ILC2 were gated on CD45^+^CD3^-^CD5^-^RORγt^-^GATA-3^hi^ cells and ILC3 were gated on CD45^+^CD3^-^CD5^-^RORγt^+^ cells. The analysis of ILC3 subsets were supported by the expression of NKp46 and CCR6. Numbers adjacent to boxed areas indicate relative percentage of gated populations. Representative results from 4 independent experiments. (B-C) Representative histograms show the absolute numbers of ILC subsets (B) T cells (C) in lung. (D) Lung cells were stimulated for 4h with IL-12, IL-18, IL-33, IL-23 and IL-1β and IFN-γ, IL-5, IL-13 and IL-22 expression in ILCs and T cells was assessed by intracellular cytokine staining and flow cytometry. Graph represents the absolute numbers of cells. Representative results from 4 independent experiments (n = 9/group). *, P<0.05.

In mucosal tissue, the innate lymphoid cell family (ILC) has an essential role for initiation, regulation and resolution of inflammation [16, 17]. In the lung of mice, group 2 ILCs are activated in response to IL-33 following influenza infection and subsequent immune-mediated tissue damage [18]. In this context, tissue repair requires IL-23 and/or IL-1β signaling, which promotes a wave of IL-22 production by group 3 ILC and T cells [19]. Moreover, the functional groups 1 ILC appear to be essential for the early phase of influenza infection [20]. As *L*. *paracasei* feeding, before or after influenza infection, induced secretion of IL-1β and IL-33 (Fig 2), we focused our attention on the presence of cytokine-expressing ILCs and T cells. ILCs were identified as CD3^-^ cells (Fig 4A). To distinguish the different ILC subsets, we combined transcription factor profiles and cell surface markers analysis to differentiate the group 1, 2 and 3 ILCs (Fig 4A) [16, 21]. Analysis of ILC subsets confirmed the unspecific expansion of immune cells in the lungs induced by *L*. *paracasei* feeding (Fig 4B). Indeed, we found a higher number of lung ILC1, ILC2 and ILC3 in *L*. *paracasei*-treated mice compared to control treated mice before the influenza infection. However, ILCs cell numbers and the cytokine releases were unaffected in both the control and *L*. *paracasei*–fed mice after viral infection except for ILC3 numbers and ILC1-secreted interferon γ (Fig 4D). Further examination of lung T cells (CD3^+^CD5^+^ cells; Fig 4C) showed no increase of IL-22 expression in *L*. *paracasei*-fed mice after 10 days of viral infection (Fig 4D). However, these cells did express a higher amount of the T helper type 2 (T_H_2) cell–associated cytokines IL-5 and IL-13 at day 10 of viral infection in *L*. *paracasei*-administrated mice (Fig 4D). We concluded that 7 days of *L*. *paracasei* feeding before infection changed the presence of ILC subsets but not cytokine expression, and generated more IFN-γ producing ILC1 (mainly NK cells) and T_H_2 cells during the late phase of influenza infection.

### Effect of *L*. *paracasei* ingestion on gut microbiota composition

**Fig 5:**
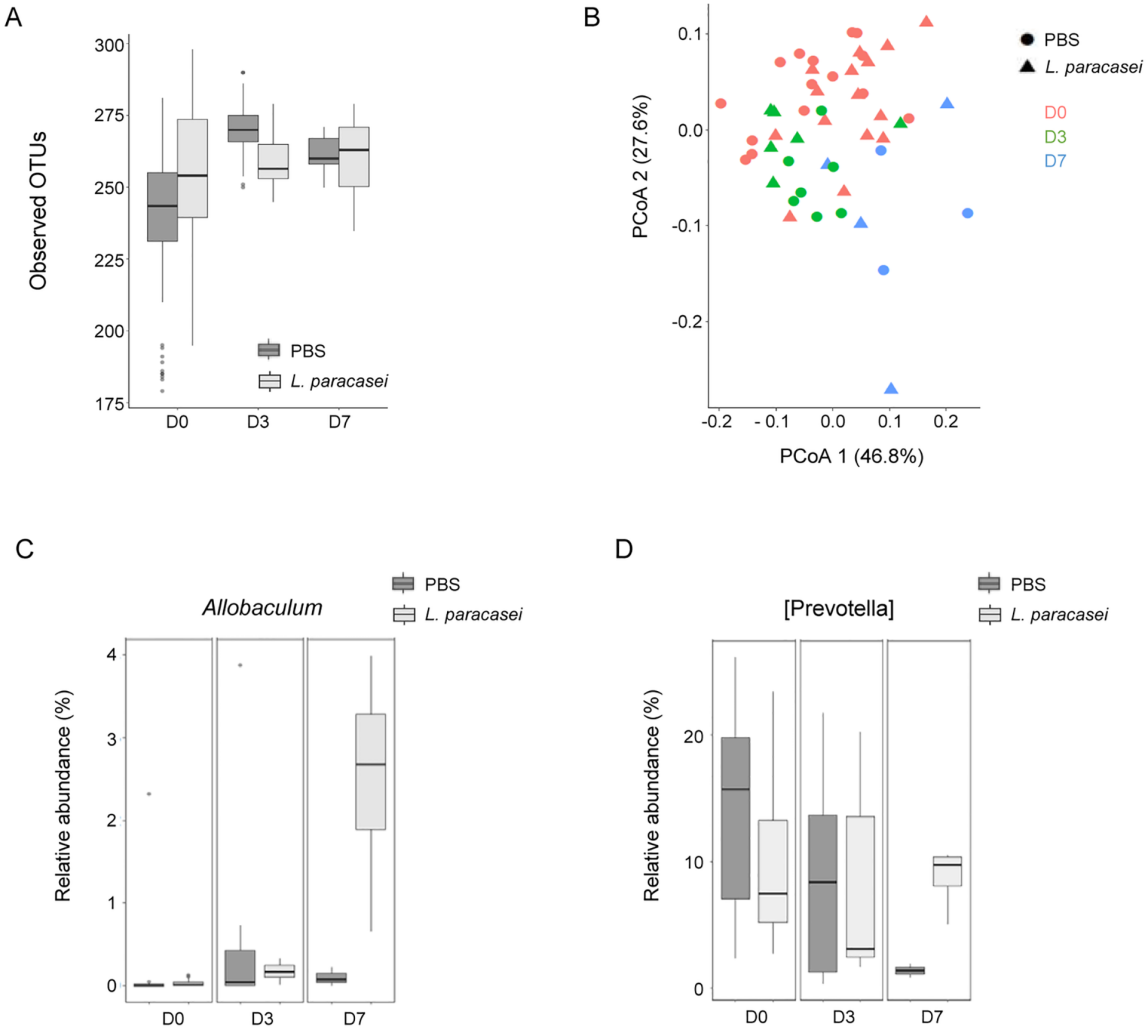
Impact of consumption of *L*. *paracasei* CNCM I-1518 on gut microbiota. (A) Alpha-diversity measured by number of OTUs before and after influenza infection. (B) Weighted Unifrac PcoA before and after infection infection (D0, D3 and D7). (C) Relative abundance (%) of *Allobaculum* through the experiment. (D) Relative abundance (%) of *Prevotella* through the experiment.

To decipher whether the observed effect of *L*. *paracasei* on health improvement and immunity could be linked to an indirect effect via the modulation of gut microbiota, feces samples were collected at different time points of influenza infection. Using alpha and beta-diversity metrics, number of OTUs and weighted Unifrac distance respectively, we did not observe any difference in microbiota composition at D-7 between both groups suggesting that both groups were homogeneous in term of microbiota before any treatment (S7 Fig). We next investigated whether 7-day ingestion of *L*. *paracasei* CNCM I-1518 affected gut microbiota before influenza infection in comparison to that of PBS-fed control mice. Following 7 days ingestion of *L*. *paracasei* or PBS the gut microbiota was not modified when looking at alpha (Fig 5A), beta–diversity using Unifrac distance (Fig 5B).

We next investigated whether consumption of *L*. *paracasei* modulated gut microbiota in influenza-infected mice 3 and 7 days post-infection. Using Weighted Unifrac distance, we observed a gradual change in gut microbiota (Fig 5B), which can be related to both infection and time effect (p = 0.003). However, this effect was independent to treatment group, suggesting that *L*. *paracasei* fed mice did not induce differential microbiota after influenza infection. Microbiota richness was increased in PBS-treated mice only 3 days post infection, while no difference was observed in *L*. *paracasei*-fed mice. However, no difference was observed 10 days post infection. Regarding microbiota composition at genus level, two genera were significantly modulated between both treatments, but only 7 days post influenza infection. *Allobaculum* was significantly increased 7 days post infection in *L*. *paracasei*-treated group compared to PBS-treated group (Fig 5C), while a genus related to *Prevotella* significantly decreased in PBS-treated group (Fig 5D). Overall, these results suggest that *L*. *paracasei* did not strongly modulate gut microbiota before and after influenza infection.

### Effects of *L*. *paracasei* peptidoglycans on influenza infected mice

**Fig 6:**
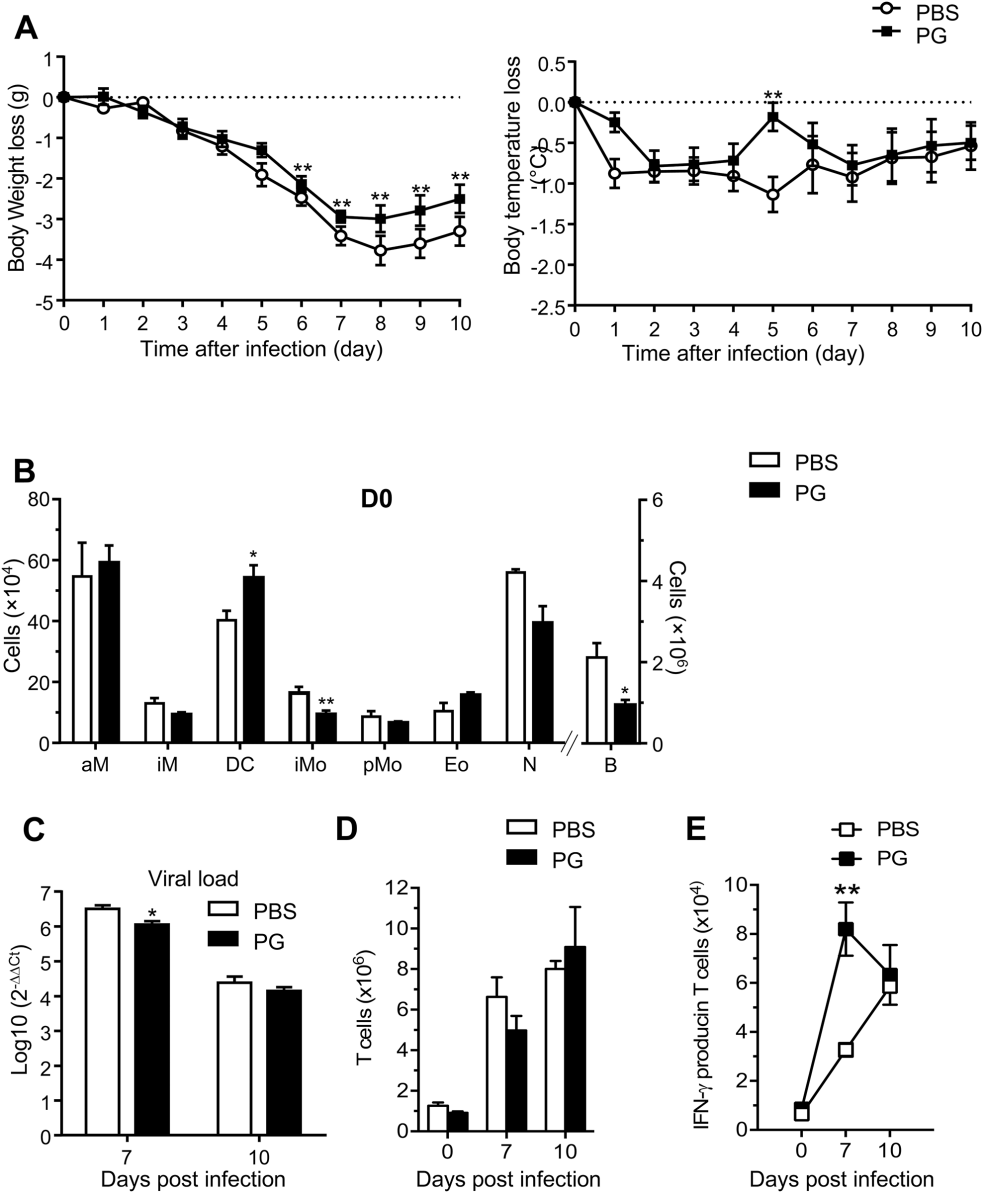
Effect of *L*. *paracasei* derived-peptidoglycans on influenza_infected mice. (A) Effects of consumption of PG of *L*. *paracasei* by mice (n = 17 at D0) on body weight loss and temperature loss compared with PBS control group (n = 17 at D0). (B) Effect of consumption of PG of *L*. *paracasei* (n = 10) on the recruitment of immune cells in the lungs at day 0 compared with the control group (n = 10): aM: Alveolar Macrophage, iM: Interstitial Macrophage, DC: Dendritic cells, iM: Inflammatory Monocyte, pM: Patrolling monocytes, Eo: Eosinophils, N: Neutrophils, B: B cells, T: T cells. Results are expressed as mean ± SEM for each group. (C) Effect of consumption of PG of *L*. *paracasei* (n = 17 at D0) on viral load in IAV-infected BALB/c mice compared with the control group (n = 17 at D0), 7 and 10 days after viral infection. Data are means ± SEM of each group of mice (*p<0.05).

To address whether bacterial components could recapitulate *L*. *paracasei* protective effects, mice were fed with purified peptidoglycan of *L*. *paracasei* CNCM I-1518. We selected a dose equivalent to 1 mg peptidoglycan per gavage, since this exceeded the peptidolgycan dose previously found to induce anti-inflammatory immune responses in a mouse model, using a *Lactobacillus salivarius* strain [13]. Health status improvement was observed, but to a much lesser extent than with the whole bacterial strain. Body weight loss and temperature loss were also slightly less pronounced in the *L*. *paracasei* peptidoglycan-fed mice, compared to the control group (Fig 6A). Gavage with peptidoglycan triggered a significant reduction of viral loads in the lungs, 7 days after infection (Fig 6C). Regarding immune cells recruitment in the lungs after 7 days of gavage, prior to influenza infection, a significant increase was observed for dendritic cells but not for other cell types (Fig 6B); there was actually a decrease of interstitial macrophages and of the B cell population. Thus, oral administration of *L*. *paracasei* peptidoglycan partially induced the protective effects of the entire *L*. *paracasei* bacterium.

## Discussion

Our results show that consumption of *L*. *paracasei* CNCM I-1518 improve the health status of mice infected by influenza, this general improvement manifested mainly by reduced weight loss and improved appearance evolution in the group fed with *L*. *paracasei*. These data are in agreement with the clinical observations and other previously reported data obtained [22–24].

It was previously described that influenza virus could induce intestinal injuries even after intranasal injection [25, 26]. One could hypothesize that *L*. *paracasei*-induced health improvement could be due to a local protection from influenza-induced intestinal damages. Nevertheless, in our model, no histological lesions were evidenced in the small intestines of influenza-infected mice compared to control Balb/c mice fed with PBS. However, the viral strain and host genetic background could be important pathogenic factors [25, 26].

In this report, we provided an extensive characterization of the modifications in the innate immune response associated with *L*. *paracasei* administration. *L*. *paracasei* consumption seems to allow an early activation of pro-inflammatory cytokines (IL-1α, IL-1β) and a massive recruitment of immune cells in the lungs after *L*. *paracasei* gavage and prior to influenza infection. IL-1α and IL-1β are induced in response to oxidative, inflammatory or metabolic stressors. This initial IL-α/β-dependent chemokine production leads to the recruitment of myeloid cells to the stressed tissue [27] Thus, we propose that *L*. *paracasei* induces an early induction of an inflammatory response triggered by the passive release of IL-1α/β into the lung tissue as an alarm. This pre-activation state of the immune system seems to be responsible of the faster clearance of influenza virus, confirmed by the rapid expansion of IFN-γ producing ILC1 (NK cells essentially). The lower inflammatory response observed after a viral infection in *L*. *paracasei*-fed mice may explain the better health state compared to control-fed mice. Interestingly, we also observed that IL-33 was detected at a significantly higher level at D7 in the lungs of mice *L*. *paracasei*–fed. IL-33 has an essential role in inducing ILC2 and T_H_2 responses [28]. Published studies have demonstrated that the T_H_2 production of the cytokine IL-13, in response to stimulation with IL-33, can promote the hyperplasia of epithelial cells in the context of airway inflammation [29, 30]. Here *L*. *paracasei*–fed mice showed an expansion of T_H_2 cells after viral infection, which suggest that IL-13 and IL-5 produced by T cells have beneficial effects for the tissue homeostasis. Although lung resident ILC2 were able to produce large amounts of IL-5 and IL-13, these cells (ILC2) might have not a prominent role in this viral context. It remains unclear whether IL-22 producing ILC3 and T cells are implicated in the tissue repair in our model, or whether they are implicated in the early phase of the viral infection in *L*. *paracasei*-fed mice. Finally, 10 days after viral infection, we observed more of the anti-inflammatory cytokine, IL-10, in the lungs of *L*. *paracasei*-fed mice compared to those gavaged with PBS. This observation supports our hypothesis of an early beneficial induction of the pro-inflammatory response before influenza infection and a lower inflammatory response after this infection to counteract an overactive immune response.

Our results suggest that *L*. *paracasei*-induced signals influence the immune response at distal site. However, the mechanism by which this occurs remains unclear. To investigate whether this effect could be mediated by bacterial component(s), *L*. *paracasei* peptidoglycan was orally administered to mice. Peptidoglycan is a major component of bacterial wall that was already shown to be involved in the induction of the immune response in the lungs of Balb/c mice [31]. Peptidoglycan is able to partially reproduce the effect of entire *L*. *paracasei* bacteria. In particular, dendritic cells recruitment in the lungs at D0 after peptidoglycans gavage may help controlling viral infection as suggested by the reduction of viral load at D7 of flu infection. Further work will be required to understand which other bacterial components or secreted metabolites such as Short Chain Fatty Acids promote additional modifications that are involved in the health improvement. Indeed, it has been described that acidic exopolysaccharide from *L*. *bulgaricus* was able to confer protection against influenza in mice [32]. We also investigated whether the impact of *L*. *paracasei* on influenza infection could be indirect, via modulation of the gut microbiota. Indeed increased amount of studies observed a relation between gut microbiota members and respiratory tract, the so-called gut-lung axis [33, 34]. At homeostasis, after 7 days of gavage without infection, we did not observe significant differences in the microbiota composition between *L*. *paracasei* and control-fed mice. Interestingly, we observed that 7 days post infection, two bacterial genera were differently modulated between *L*. *paracasei* and control groups. *Allobaculum* was significantly increased while an unclassified [*Prevotella*] significantly decreased following *L*. *paracasei* gavage. Interestingly, Cox et al [35] observed that *Allobaculum*, positively correlated with RORγt and IL-17 expression, suggesting roles in intestinal Th17 differentiation in a low-dose penicillin murine model.

In summary, the results presented in our report here bring an experimental support for the clinical improvement of influenza infection that was observed in children, stressed adults and elderly who daily consumed a fermented dairy product containing the same *L*. *paracasei* CNCM I-1518 strain [6, 11]. Moreover, our data also provide evidences that the cell-mediated responses contribute to protection against influenza A virus. Such a mechanism can now be further explored as complementary adjuvant management of flu infection that should help alleviate the clinical severity of flu infection and improve health outcomes.

## Methods

### Ethical statement

Animal work in this study was carried out at the Institut Pasteur in strict accordance with the European Union Directive 2010/63/EU (and its revision 86/609/EEC) on the protection of animals used for scientific purposes. The laboratory at the Institut Pasteur has the administrative authorization for animal experimentation (Project Number 75–1554) and the protocol was approved by the Institut Pasteur Review Board that is part of the Regional Committee of Ethics of Animal Experiments of Paris Region (Permit Number: 99–174 and 2013–0109).

### Mice

Six-week old female BALB/c mice were purchased (Janvier, Genest-Saint-Isle, France) and kept one week prior to the experiment in biosafety containment animal facility. All mice were housed under specific pathogen free condition at the Institut Pasteur, provided with food and water ad libitum. The mice were euthanized by injection of high dose of chemical anesthetics (pentobarbital).

### Bacterial and viral strains

*L*. *paracasei* CNCM I-1518 was cultured in MRS (Man, Rogosa, Sharpe) broth (DIFCO^®^) at 37°C overnight in aerobic conditions. The cultured bacteria were harvested by centrifugation (4500 g) and washed with sterile PBS (Gibco). After washing, the optical density was measured at 600 nm (OD_600nm_) and adjusted with additional PBS to obtain a final OD_600nm_ of 1. The bacterial suspension was stored at -80°C in aliquots until use. Influenza A virus, A/Scotland/20/74 (H3N2); IAV) adapted to mice was made from lung homogenates in 30% glycerol and stored at -80°C. The virus was prepared, stored in aliquots at -80°C and thawed prior to infection as previously described [12].

### Probiotic treatment and influenza infection

Mice were orally gavaged (200 μl) with *L*. *paracasei* CNCM I-1518 (2x10^8^ CFU) provided by Danone Research or PBS (control) daily for 7 days before infection (Fig 1A). At D0, mice were anesthetized with sodium pentobarbital (Sanofi, Santé Animale, Libourne France) and infected intranasally with 50 μl of virus H3N2 (260 plaque-forming units, pfu), as previously described [12]. Mice were followed up to 10 days after viral infection and *L*. *paracasei* oral gavage was continued during the whole period of viral infection. Health status and appearance score of mice were followed during the whole experiment (17 days). Weight, coetaneous temperature (using Infrared Thermometer from Bioseb) and fur appearance of each mouse were measured daily. Results were expressed as body weight loss and temperature loss compared to values for each mouse at D0. Fur appearance was scored after infection at D0 as follows: 3; Coat is smooth, 2; Patches of hair piloerected, 1; Majority of back is piloerected, and 0; Piloerection may or may not be present, mouse appears “puffy”[36]. Mice were sacrificed at different time points of the experiment (n = 5 mice sacrificed at each time point/ experiment, unless otherwise mentioned). After perfusion, lungs were extracted, homogenized in order to eliminate heterogeneity of lesions distribution and then divided into three lots and used to perform the cytokine assay, flow cytometry analysis and RT- real time PCR.

### Quantification of influenza A virus

Lung tissues were homogenized and RNA was isolated with an RNeasy micro kit (Qiagen) according to the manufacturer’s instructions, then cDNA was generated using AMV reverse transcriptase (Promega). An ABI7300 real-time PCR (Applied Biosystems) was used for real-time quantitative PCR analysis of cDNA with primers generated to be specific for the sequence encoding the M2 protein of IAV. Samples were normalized to Ribonuclease P expression. Viral load was determined by relative quantification using the 2^(-ΔΔC(T))^ method [37]. The protocol was realized according to WHO recommendations.

(http://www.who.int/influenza/gisrs_laboratory/cnic_realtime_rt_pcr_protocol_a_h7n9.pdf). Viral load were also inferred from standard range realized by serial dilution of homogenized lung infected with a known concentration of H3N2 virus.

### Detection of *L*. *paracasei* CNCM I-1518 in the lung

The presence of living *L*. *paracasei* was performed by culture of lung suspensions on MRS agar plates 24h or 48h after oral gavage with *L*. *paracasei*. Additionally, presence of *L*. *paracasei* DNA was detected by specific PCR targeting CRISPR (clustered regularly interspaced short palindromic repeats) of *L*. *paracasei* strain [38].

### Histology

Following sacrifice of mice, small intestines or lungs were removed and fixed immediately in 10% neutral-buffered formalin in PBS for 24 hours, embedded in paraffin and cut into 4 μm or 5 μm sections. After deparaffinization, sections were stained with hematoxylin and eosin (HE) before scanning with a light microscope (Nanozoomer2.0, Hamamatsu). All images were obtained at X20 magnification and analyzed with NDPview software (Hamamatsu).

### Determination of cytokine levels

Various cytokines (Mip-1α, Mip-1β, IFN-γ, MCP-1, IL5, IL10, IL1α, IL1β and IL33) were quantified in the cleared lung homogenate from mice scarified on day 0, 7 and 10, using Magnetic Luminex Screening Assay (R&D systems, Minneapolis City, USA) according to the manufacturer’s instructions. The cytokine levels in the lungs were expressed as the amount of cytokine per unit weight of total protein of the lung. The quantification of total protein was determined using BSA protein Assay Kit (Thermos scientific, Meridian Road, USA) according to the manufacturer’s instructions.

### Antibodies and flow cytometry

Single-cell suspensions were prepared from the lungs that were mechanically disrupted using collagenase IV (Sigma-Aldrich, St. Quentin Fallavier, France) as previously described [39]. Red blood cells were lysed using ACK buffer (Gibco).

Cell-surface molecule staining, transcription factor and cytokine intracellular staining were performed as previously described [40]. Flow cytometry data were collected with the LSR Fortessa (BD Biosciences) and the results were analyzed with FlowJo software (v10; Tree Star). Gating strategies are depicted in Fig 4A and S8 Fig. Antibodies specific to mice were purchased from BD Biosciences, Biolegend and eBioscience.

### Preparation of *L*. *paracasei* peptidoglycan

Bacterial cultures were grown overnight to stationary phase in MRS broth, and peptidoglycan purified as described previously [41]. Briefly, sacculi were extracted by boiling in SDS (4% wt/vol) for 1 hour, washed by resuspension with MilliQ (MQ)-H_2_O and centrifugation for 30 min at 40,000 ×g until free of SDS (determined using the method described in [42]. Cells were broken by mechanical shearing with glass beads (Sigma Aldrich) using a Precellys24 homogenizer (Bertin Instruments). Cell walls were resuspended in 50 mM Tris pH 7 and sequentially digested at 37°C with α-amylase (100 μg/ml; overnight), DNase (10 μg/ml) and RNase (50 μg/ml) plus 20 mM MgSO_4_ (2 hours) and trypsin (100 μg/ml) plus 10 mM CaCl_2_ (overnight). Following boiling for 15 min in 1% SDS, the cell pellets were washed once by resuspension with MQ-H_2_O and centrifugation as before. Pellets were incubated for 15 min at 37 with 8M LiCl °C, followed by 100 mM EDTA pH 8, and then washed twice with MQ-H2O by resuspension-centrifugation as before. The pellet was resuspended in acetone by sonication, then the cell pellet collected, washed twice with MQ-H2O by resuspension-centrifugation, and the white pellet dried by speed-vac, then resuspended in 48% hydrofluoric acid (HF) for 48 hours. HF was removed by extensive resuspension and centrifugation with MQ-H2O until the pH was stable at around 5. The purified peptidoglycan was dried then resuspended to 5mg/ml with 12.5 mM sodium phosphate buffer pH 5.6 and digested overnight with 100 units/ml mutanolysin (Sigma). Reagents were prepared using endotoxin free water. The reaction was stopped by boiling for 3 min. No undigested material was observed, suggesting the peptidoglycan was fully solubilized. Mice were gavaged with 200 μl volumes of the resulting solution, equivalent to 1 mg *L*. *paracasei* peptidoglycan.

### Gut microbiota composition analysis by 16S sequencing

80 fresh fecal samples were collected from *L*. *paracasei* and PBS- gavaged mice at several time points. Pellets were stored at −80°C until extraction. Fecal DNA was extracted using mechanical lysis (Fastprep^®^ FP120 (ThermoSavant) followed by phenol/chloroform-based extraction as previously described [43]. Gene specific primers for the bacterial 16S rRNA were used to amplify the V3-V4 region (341F-806R) following the protocol from [44]. The library pool was mixed with 10% of Illumina PhiX control libraries. Mixed Phix/16S libraries were sequenced in multiplex on the MiSeq machine with the MiSeq v3 chemistry to perform paired-end 300bp sequencing. The paired-end reads were assembled with FLASH [45] (default parameters). Quality trimming and filtering (quality and length based) have been realized with QIIME (v1.8) [46]. Chimeric sequences have been predicted *de novo* with uchime [47] which is integrated in the usearch6.1. QIIME pipeline version 1.9, 97% d*e novo* OTUs were picked using vsearch algorithm (version 2.0.3). RDP classifier trained on GreenGenes database (version 13.8, august 2013) [48] was used for the taxonomic OTU annotation. A second level of sequence filtering based on OTU read counts (OTU read count> 0.005% of total read count) was performed as recommended in [49]. After quality filtering, a total 2,481,960 assembled reads, 31024 ± 8488 / sample, were included for downstream analyses. For alpha- and beta-diversity analyses, samples were rarefied to 15,000 sequences per sample. Alpha-diversity (that measures diversity within samples) was assessed using rarefaction curves for richness (Observed OTUs), and evenness (Shannon index). Beta-diversity analysis (that measures diversity between samples) was performed on weighted Unifrac distances. Raw counts were normalized with the DESeq2 Bioconductor package normalization function [50]. Normalized counts were converted to log2(CPM), counts per million (voom function, limma package) [51]. Paired statistical analysis of the transformed data was performed with limma (block factor: mouse). Benjamini-Hochberg method [52] was used for p. value adjustment for multiple testing, (FDR threshold: 0.05). An additional filter was used to filter significant OTU/taxa: at least 3 non-zero counts, in at least one of the groups considered in the contrast. Global comparison of groups was performed using permutational anova [53].

### Statistical analysis

Data were expressed as means ± S.D or means ± SEM. Student’s t tests were used to analyze group differences. These analyses were conducted using PRISM 6.04 (GraphPad).

In addition, body weight and temperature kinetics (D1 to D10) were analyzed using linear mixed models for repeated measures adjusted by baseline value (D0) using SAS 9.3.

Values of p<0.05 were considered significant.

## Supporting information

S1 Fig This S1 Figure and its legend. (PDF)

S2 Fig This S2 Figure and its legend. (PDF)

S3 Fig This S3 Figure and its legend. (PDF)

S4 Fig This S4 Figure and its legend. (PDF)

S5 Fig This S5 Figure and its legend. (PDF)

S6 Fig This S6 Figure and its legend. (PDF)

S7 Fig This S7 Figure and its legend. (PDF)

S8 Fig This S8 Figure and its legend. (PDF)
