# Supplementary material for: Lactobacillus paracasei feeding improves immune control of influenza infection in mice
Source: PLoS One. 2017 Sep 20;12(9):e0184976. doi: 10.1371/journal.pone.0184976 (PMC5607164; doi:10.1371/journal.pone.0184976)
Supplement: S1 Fig — (PDF) [file pone.0184976.s001.pdf]

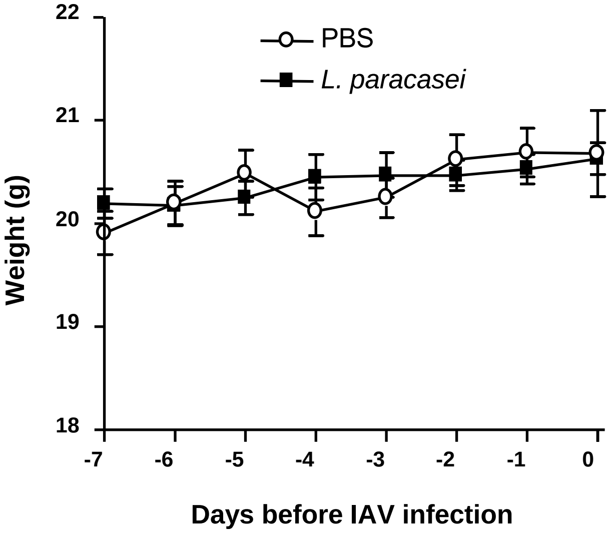

S1 Figure. Mice weight prior to influenzae infection. (B) Body weight of mice gavaged with *L. paracasei* CNCM I-1518 strain or PBS between D-7 and D0 (before IAV infection). Body weights of individual mice were expressed in gram as a mean of mice in each group ±SEM.
