# Supplementary material for: Lactobacillus paracasei feeding improves immune control of influenza infection in mice
Source: PLoS One. 2017 Sep 20;12(9):e0184976. doi: 10.1371/journal.pone.0184976 (PMC5607164; doi:10.1371/journal.pone.0184976)
Supplement: S2 Fig — (PDF) [file pone.0184976.s002.pdf]

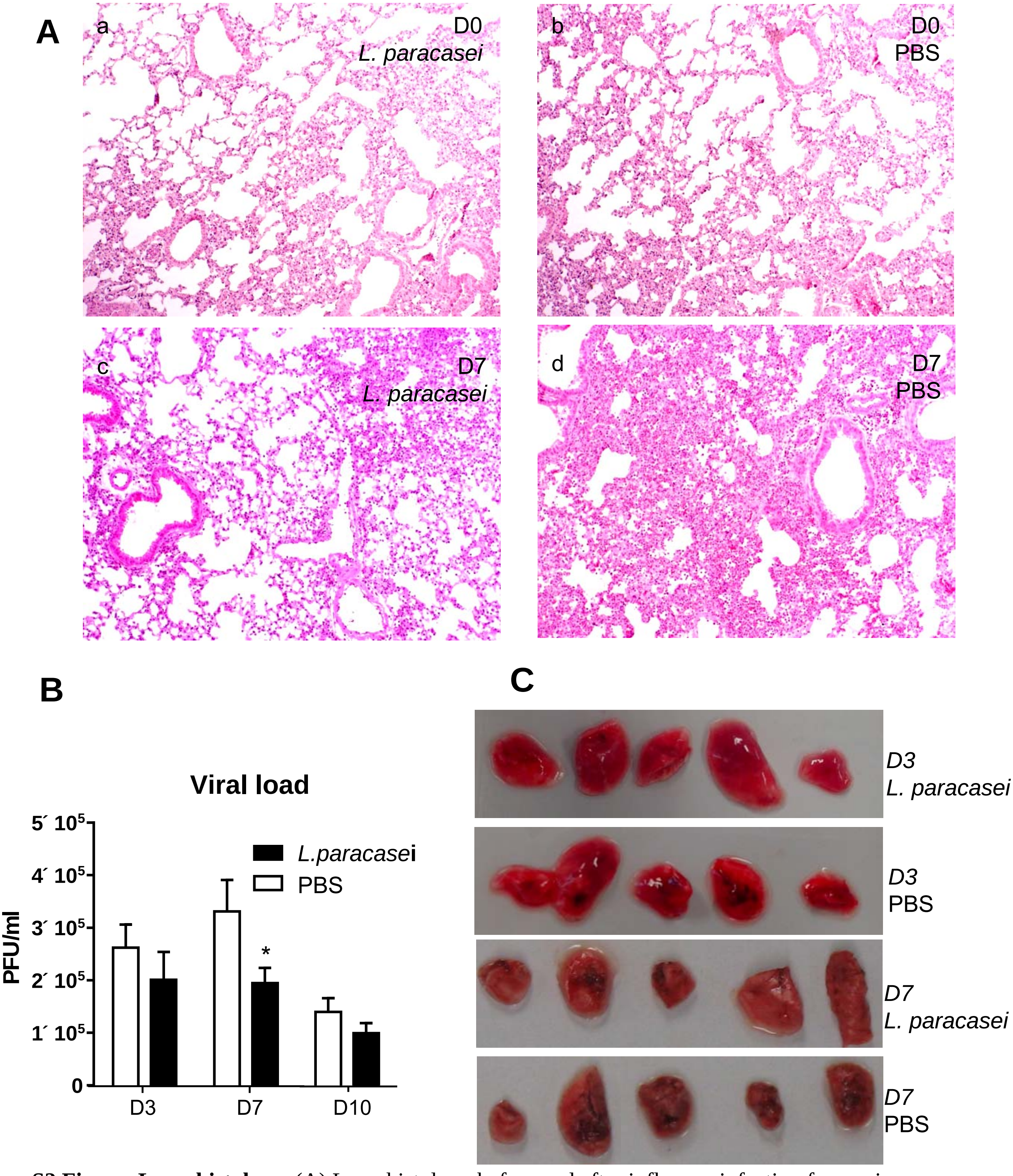

S2 Figure. Lung histology. (A) Lung histology before and after influenza infection from mice gavaged with either PBS or *L. paracasei* at day 0 (a and b respectively) and at day 7 post-infection (c and d respectively). (B) Hemorrhagic lesions in the lungs of *L. paracasei*- or PBS fed mice at day 3 and at day 7 post influenza infection. (C) Viral load measured by real time PCR at D3, D7 or D10 post influenza infection in lungs of mice fed with either *L. paracasei* (N=18) or with PBS (N=18).
