# Supplementary material for: Lactobacillus paracasei feeding improves immune control of influenza infection in mice
Source: PLoS One. 2017 Sep 20;12(9):e0184976. doi: 10.1371/journal.pone.0184976 (PMC5607164; doi:10.1371/journal.pone.0184976)
Supplement: S3 Fig — (PDF) [file pone.0184976.s003.pdf]

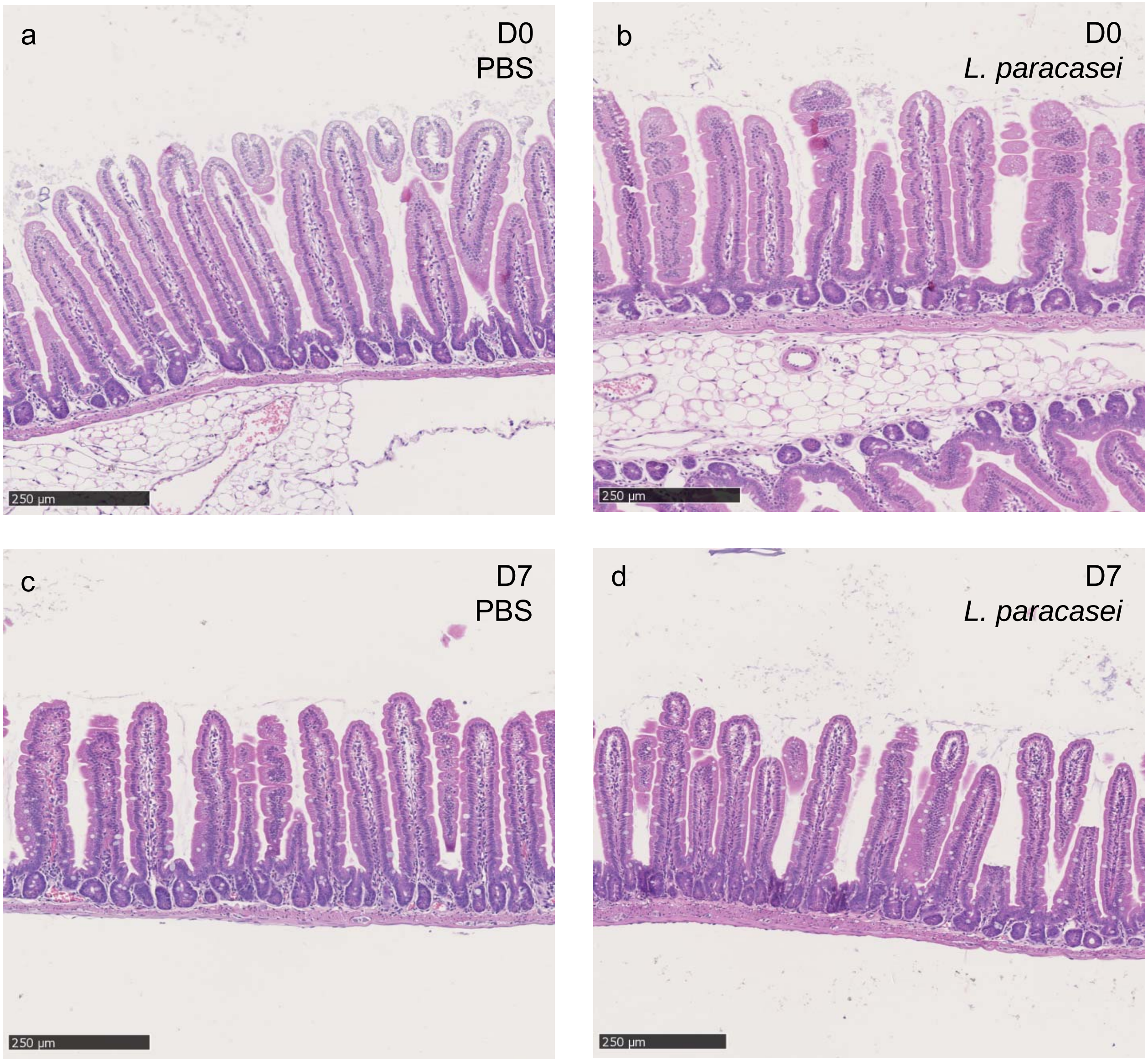

S3 Figure. Gut histology before and after influenza infection. HE-stained sections of small intestine (jejunum region) from mice gavaged with either PBS or *L. paracasei* at day 0 (a and b respectively) and at day 7 post-infection (c and d respectively). Data are representative of three independent experiments N=3/group. Scale bar: 250 µm.
