# Supplementary material for: Lactobacillus paracasei feeding improves immune control of influenza infection in mice
Source: PLoS One. 2017 Sep 20;12(9):e0184976. doi: 10.1371/journal.pone.0184976 (PMC5607164; doi:10.1371/journal.pone.0184976)
Supplement: S4 Fig — (PDF) [file pone.0184976.s004.pdf]

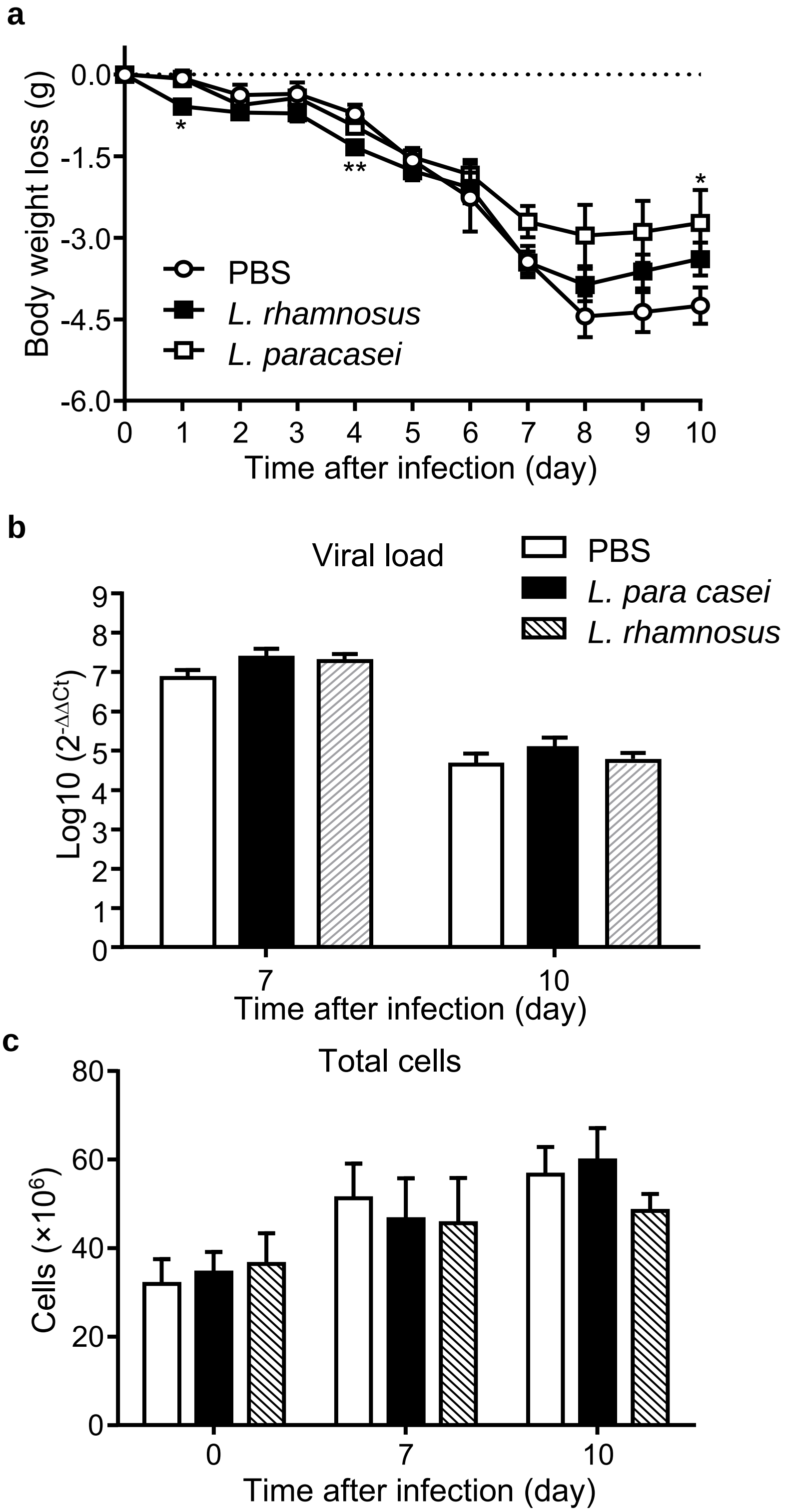

S4 Figure. Effects of 2 others lactobacilli strains on influenza-infected mice. (A) Body weight loss was expressed as difference between values at each time point after flu infection compared to weight at D0. The mice tested belonged to three groups: one group was fed with PBS, the second group was fed with *L. paracasei* CNCM I-3689 and the third group was fed with *L. rhamnosus* CNCM I-3690 (N = 20 in each groups) (B) Viral load measured with RTq-PCR at D7 or D10 post influenza infection in lungs of mice fed with either PBS, *L. paracasei* CNCM I-3689 or *L. rhamnosus* CNCM I-3690. N= 10 in each groups (C) Total cells counts in the lung cell suspensions quantified before (day 0) and after influenza infection (days 7 and 10), using trypan blue, N= 20 in each groups (*L. paracasei* CNCM I-3689, *L. rhamnosus* CNCM I-3690 or PBS groups).
