# Supplementary material for: Lactobacillus paracasei feeding improves immune control of influenza infection in mice
Source: PLoS One. 2017 Sep 20;12(9):e0184976. doi: 10.1371/journal.pone.0184976 (PMC5607164; doi:10.1371/journal.pone.0184976)
Supplement: S5 Fig — (PDF) [file pone.0184976.s005.pdf]

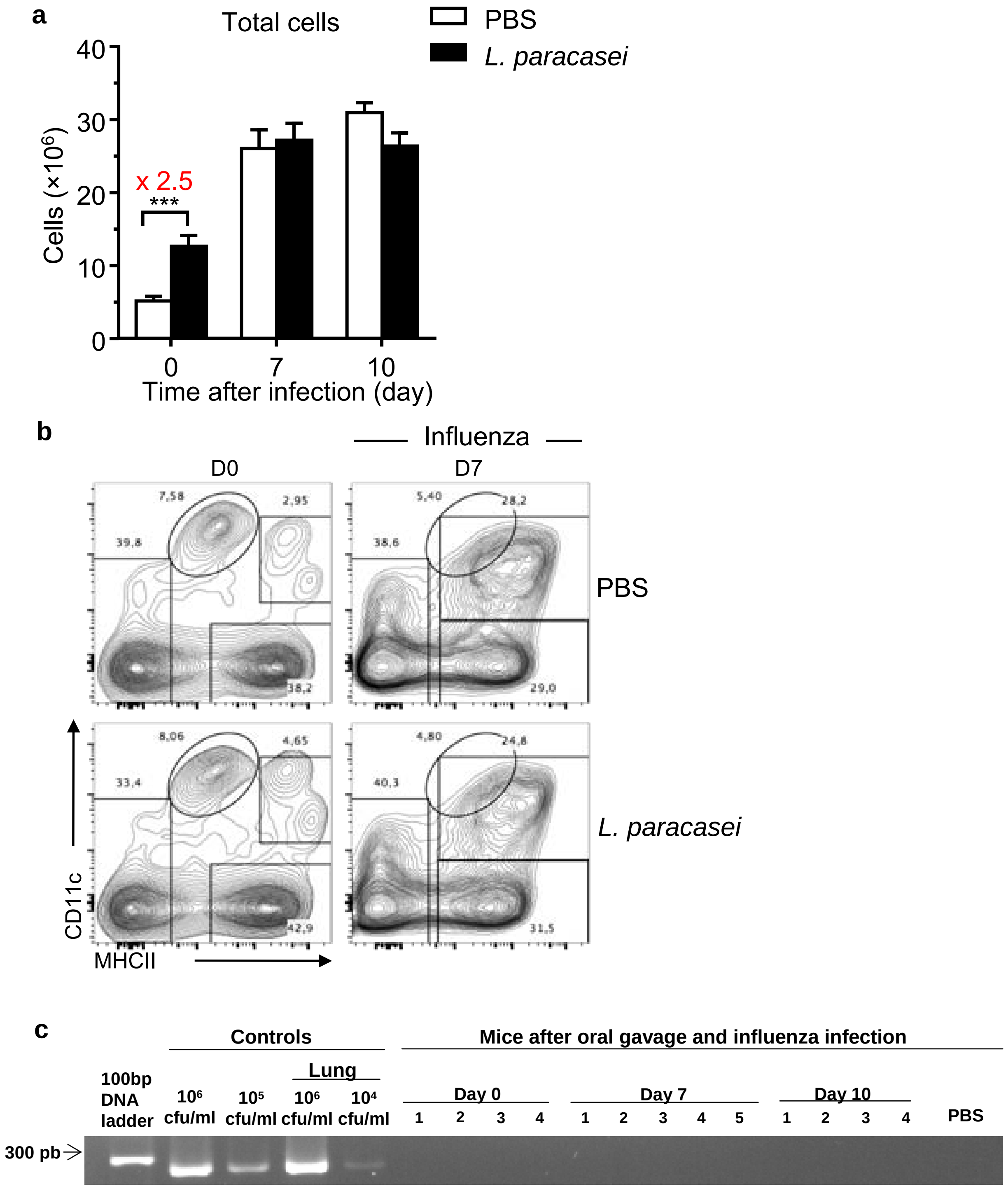

S5 Figure. Effect of consumption of *L. paracasei* on immune cells in mice lungs. (a) Total cells counts in the lung cell suspensions quantified before (day 0) and after infection (days 7 and 10), using trypan blue, N=30 in each groups (*L. paracasei* or PBS group); (b) Myeloid cells profile in flow cytometry analysis (using CD11c and MHCII labeling), comparison between *L. paracasei* gavaged mice and PBS gavaged mice Before and after flu infection. (c) PCR targeting CRISPR (clustered regularly interspaced short palindromic repeats) specific of *L. paracasei* strain, a positive control for DNA was used by contamination of a spiked lung suspension with known *L. paracasei* concentrations.
