# Supplementary material for: Lactobacillus paracasei feeding improves immune control of influenza infection in mice
Source: PLoS One. 2017 Sep 20;12(9):e0184976. doi: 10.1371/journal.pone.0184976 (PMC5607164; doi:10.1371/journal.pone.0184976)
Supplement: S6 Fig — (PDF) [file pone.0184976.s006.pdf]

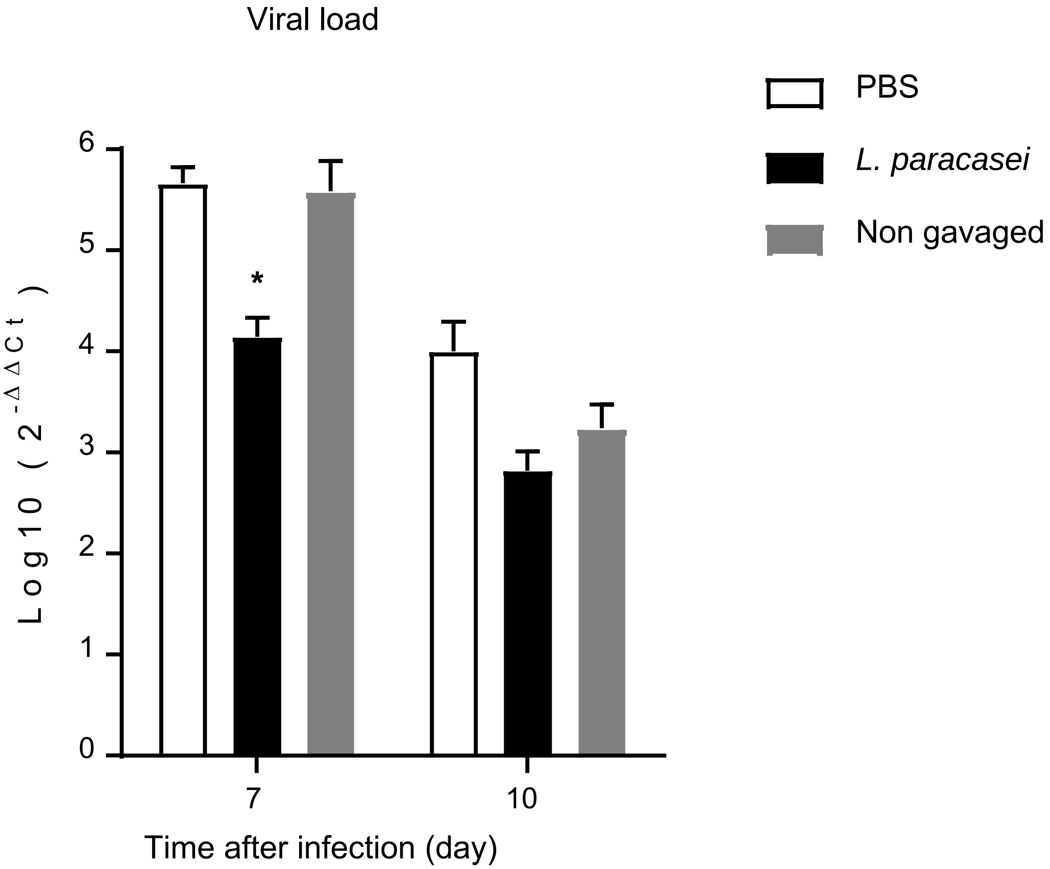

S6 Figure. Effect of gavage viral load in mice lungs. Three groups of mice were used (n=10 mice per group): one group was not gavaged, one group was gavaged with PBS and the third group was gavaged by *L. paracasei* CNCM I-1518 strain. Gavage was continued for 7 days then all mice were flu-infected and followed for 10 days under the same conditions regarding the gavage. Five mice from each group were sampled at day 7 day 10 after flu infection. IAV-viral load measured by RT real time-PCR in lungs of mice that were not gavaged or fed with either PBS or *L. paracasei*. Star indicates significant difference (P<0.05)
