# Supplementary material for: Lactobacillus paracasei feeding improves immune control of influenza infection in mice
Source: PLoS One. 2017 Sep 20;12(9):e0184976. doi: 10.1371/journal.pone.0184976 (PMC5607164; doi:10.1371/journal.pone.0184976)
Supplement: S7 Fig — (PDF) [file pone.0184976.s007.pdf]

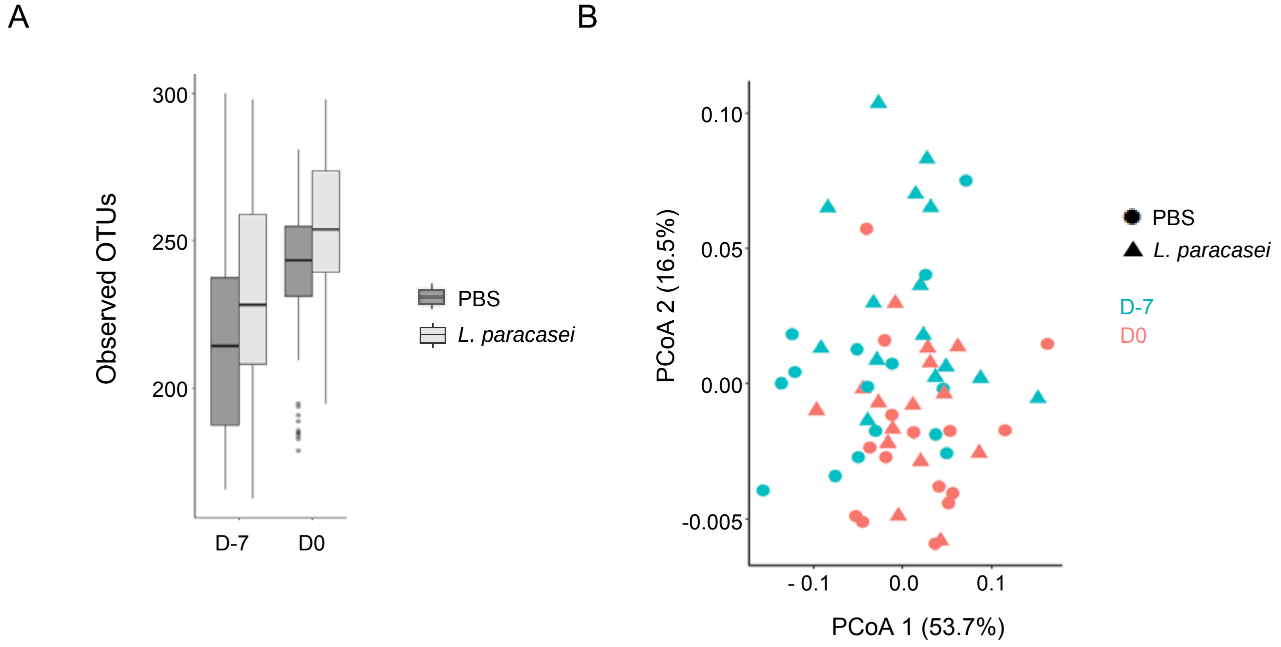

S7 Figure. Impact of consumption of *L. paracasei* CNCM I-1518 on gut microbiota before influenza infection. (A) Alpha-diversity measured by number of OTUs before infection (D-7 and D0). (B) Weighted Unifrac PcoA before infection (D-7 and D0).
